# Combined Effect of Melittin and DNase on *Enterococcus faecalis* Biofilms and Its Susceptibility to Sodium Hypochlorite

**DOI:** 10.3390/ma13173740

**Published:** 2020-08-24

**Authors:** Sujitha Ramaraj, Mi-Ah Kim, Vinicius Rosa, Prasanna Neelakantan, Won-Jun Shon, Kyung-San Min

**Affiliations:** 1 Department of Conservative Dentistry, School of Dentistry and Institute of Oral Bioscience, Jeonbuk National University, Jeonju 54896, Korea; sujitharamaraj@yahoo.in (S.R.); miah2018@hanmail.net (M.-A.K.); 2 Discipline of Oral Sciences, Faculty of Dentistry, National University of Singapore, Singapore 119085, Singapore; denvr@nus.edu.sg; 3 Discipline of Endodontology, Department of Restorative Dental Sciences, Faculty of Dentistry, The University of Hong Kong, Hong Kong, China; prasanna@hku.hk; 4 Department of Conservative Dentistry, School of Dentistry, Seoul National University, Seoul 03080, Korea; 5 Research Institute of Clinical Medicine of Jeonbuk National University, Jeonju 54907, Korea; 6 Biomedical Research Institute of Jeonbuk National University Hospital, Jeonju 54907, Korea

**Keywords:** biofilm, DNase, *Enterococcus faecalis*, melittin, sodium hypochlorite

## Abstract

Biofilm communities are tolerant to antimicrobials and difficult to eradicate. This study aimed to investigate the effect of melittin, an antimicrobial peptide, either alone or in combination with deoxyribonuclease (DNase), an inhibitor of extracellular deoxyribonucleic acid (eDNA), against *Enterococcus faecalis* (*E. faecalis*) biofilms, and biofilm susceptibility to sodium hypochlorite (NaOCl). Biofilms of *E. faecalis* were developed in root canals of bovine teeth. The biofilms were treated with distilled water (control), melittin, DNase, or DNase+melittin. The antibiofilm effects of the treatments were analyzed using colony forming unit (CFU) assay, crystal violet staining, confocal laser scanning microscopy (CLSM), and field emission scanning electron microscope (FE-SEM). The susceptibility of DNase+melittin-treated biofilms to NaOCl (0%, 2.5% and 5%) was investigated by the CFU assay. The data were statistically analyzed using one-way analysis of variance, followed by Tukey’s test. A *p*-value of <0.05 was considered significant. Specimens treated with DNase+melittin showed a more significant decrease in the CFUs, eDNA level, and biofilm formation rate than those treated only with melittin or DNase (*p* < 0.05). CLSM analysis showed DNase+melittin treatment significantly reduced the volume of biofilms and extracellular polymeric substance compared to either treatment alone (*p* < 0.05). FE-SEM images showed a high degree of biofilm disruption in specimens that received DNase+melittin. 2.5% NaOCl in specimens pretreated with DNase+melittin showed higher antibacterial activity than those treated only with 5% NaOCl (*p* < 0.05). This study highlighted that DNase improved the antibiofilm effects of melittin. Moreover, DNase+melittin treatment increased the susceptibility of biofilms to NaOCl. Thus, the complex could be a clinical strategy for safer use of NaOCl by reducing the concentration.

## 1. Introduction

The persistence of microbes in the root canal system often leads to endodontic failures [1]. Among these, *Enterococcus faecalis* (*E. faecalis*), a gram-positive facultative anaerobic cocci, is one of the predominant bacteria associated with failed endodontic cases and can adapt to harsh environmental conditions by forming antimicrobial-resistant biofilms [1]. Therefore, recent studies have the objective to develop strategies aimed at inhibiting biofilm formation of *E. faecalis* in endodontic treatment [2,3].

Bacteria within biofilms are more resistant to antibacterial agents than their planktonic forms [4]. This is because the bacteria encased in biofilms are protected by a complex matrix of extracellular polymeric substances (EPS), which provide mechanical stability to the biofilms and prevent the entry/action of antimicrobials [5]. Extracellular deoxyribonucleic acid (eDNA), an important component of the extracellular matrix, is responsible for maintaining the stability of biofilms and preventing the penetration of antimicrobial agent through the matrix encased biofilms [6]. Deoxyribonuclease (DNase), an inhibitor of the eDNA, has been shown to digest eDNA in biofilms and reduce the adherence of *E. faecalis* in root canals [7,8]. Furthermore, DNase has also been widely used in the biomedical field to treat cystic fibrosis and cancer [9].

Sodium hypochlorite (NaOCl) is widely used as an endodontic irrigant because of its potent bactericidal activity [10]. However, the detrimental effects of high concentrations of NaOCl on host tissues [11] have resulted in attempts to develop strategies that can demonstrate effective antibiofilm activity [2,3]. These chemical adjuncts have been evaluated alone or in combination with NaOCl, thereby reducing the concentration of NaOCl [12,13,14].

Antimicrobial peptides (AMPs) are 12–50 amino acids in length, cationic and amphipathic molecules showing broad-spectrum activities against microorganisms, including Gram-positive and Gram-negative bacteria. In general, AMPs act by disrupting or perforating the bacterial membranes [15]. Amongst these, melittin, a short cationic linear AMP composed of 26 amino acid residues found in the venom of honeybee (*Apis mellifera*) [16], has potent antimicrobial activity and is widely used for arthritis and cancer therapy [17]. It has been shown to disrupt the biofilms of *Staphylococcus aureus (S. aureus)*, *Escherichia coli (E. coli)*, and *Pseudomonas aeruginosa* in a recent study [18].

However, the effects of combined treatments of DNase with melittin, and the effect of the treated biofilms to NaOCl at low concentrations, remains unknown. Therefore, this study aimed to investigate the effect of melittin alone and in combination with DNase on biofilms, using *E. faecalis* as the model organism. Further, the study investigated the susceptibility of DNase+melittin-treated biofilms to NaOCl. The null hypothesis was that combination of melittin with DNase is not effective against *E. faecalis* biofilms, and irrigation with DNase+melittin followed by 2.5% or 5% NaOCl is not more effective than the conventional irrigation with NaOCl.

## 2. Materials and Methods

### 2.1. Specimen Preparation

Experimental protocols in this study were approved by the Institutional Animal Care and Use Committee of Jeonbuk National University (CBNU 2018-084). Specimens were prepared according to the description of a previous study with minor modifications [19]. Briefly, freshly extracted single-rooted bovine central incisors, were obtained from a slaughterhouse and immersed in 1% NaOCl solution for 24 h for disinfection. Apical 5.0 mm and coronal two-thirds were removed from each tooth with a diamond saw (AEU-25, Aseptico, Woodinville, WA, USA). The root canal was enlarged using 3.1 mm carbide round bur (MANI Inc., Utsunomiya, Japan) and divided into blocks of 4 mm length. The specimens were then sectioned into cylindrical halves and irrigated with 17% ethylenediaminetetraacetic acid (EDTA) (Sigma-Aldrich, St. Louis, MO, USA) for 3 min to remove the smear layer and washed with distilled water (DW). These specimens were autoclaved at 121 °C for 20 min, and the outer surfaces were coated twice with nail polish (Innisfree; AmorePacific Co, Seoul, Korea). Then the specimens were kept in brain–heart infusion (BHI; Difco Laboratories, Detroit, MI, USA) at 37 °C for 24 h to ensure microbial contamination.

### 2.2. Bacteria and Culture Conditions

*E. faecalis* ATCC 29212 was used in this study. A single isolated colony from a BHI- agar plate (Difco Laboratories) grown from the frozen stock culture at −80 °C was inoculated into 5 mL of BHI broth and incubated at 37 °C for 24 h. The bacterial concentration was adjusted to an optical density (OD_600_) of 1 with sterile BHI broth spectrophotometrically.

### 2.3. Minimum Inhibitory Concentration (MIC) of Melittin

Broth microdilution testing was performed according to the clinical laboratory standard institute guidelines [20] to determine the MIC of melittin against *E. faecalis* ATCC 29212. An overnight grown bacterial suspension (100 μL) adjusted to 1 × 10^6^ CFU/mL was inoculated into 96-well polystyrene plate with 100 μL of melittin (ALX-162-006-M001; Enzo Life Sciences, Plymouth, PA, USA) ranging from 0.7 to 200 μg/mL. The plate was incubated at 37 °C for 24 h, and the optical density was recorded at 600 nm using a microplate reader (SPECTRO star^nano^, BMG LABTECH, Ortenberg, Germany). The bacterial suspension with BHI broth and broth alone were used as positive and negative controls, respectively. The experiments were performed in triplicates.

### 2.4. Biofilm Formation and Antibiofilm Treatment

Twenty-four specimens were placed vertically in a 24-well polystyrene cell culture plate. Each well was inoculated with 2 mL suspension of *E. faecalis* (1 × 10^6^ CFU/mL) and incubated at 37 °C for 7 days. Fresh BHI medium was replaced every second day. The purity of the cultures was confirmed by colony morphology on BHI agar. On the seventh day, the infected specimens were divided into four groups (*n* = 6/group) and immersed in 1 mL of the experimental agents for 1 h each as follows: control group: DW, melittin group: melittin (6 μg/mL in DW), DNase group: DNase (Invitrogen™ 18068015, Carlsbad, CA, USA) (1 μg/mL in DW), and DNase+melittin group: DNase (1 μg/mL in DW) + melittin (6 μg/mL in DW). After treatments, the specimens were used for various downstream analyses.

### 2.5. CFU Assay

After the treatment period, the samples were washed with DW and transferred into 1.5 mL tube containing 1 mL sterile water and sonicated (10 s pulses, twice at 20% energy level) to detach the biofilms from the root canal walls using an ultrasonic cell disrupter (VCX 130PB; Sonics and Materials, Newtown, CT, USA). An aliquot of 100 μL of each specimen was serially diluted and inoculated onto solid BHI plates and incubated at 37 °C for 24 h. Then, the CFUs were enumerated.

### 2.6. eDNA Quantification

eDNA quantification was determined according to a previous study [21]. Briefly, following treatments, the biofilms on the specimens were detached using sonication as described above. To measure the eDNA concentration, the detached biofilm was centrifuged at 10,000× *g* at 4 °C for 10 min. The collected supernatant was filtered using 0.22 μm Millipore filter (Merck-Millipore, Darmstadt, Germany) and treated with DNA-binding dye of SYBR Green I (Invitrogen, Carlsbad, CA, USA). The eDNA concentration was measured using a fluorescence microplate reader (HIDEX, Turku, Finland) with the absorbance/fluorescence emission maxima at 485/535 nm. Each experiment was performed in triplicates.

### 2.7. Crystal Violet Staining

Crystal violet staining analysis was performed to evaluate the adherent biofilms on the specimens. The infected specimens cultured with *E. faecalis* for 7 days at 37 °C were subjected to the above experimental groups. Following treatment, the root canal dentin was stained with 0.1% crystal violet (Sigma-Aldrich, St. Louis, MO, USA) for 10 min, followed by 30% acetic acid (Fisher Scientific, Fair Lawn, NJ, USA) for 10 min. Then, the acetic acid was dispensed to a sterile 96-well microtiter plate and the optical density was determined at 595 nm (μQuant, Biotek Instrument, Winooski, VT, USA).

### 2.8. Confocal Laser Scanning Microscopy (CLSM) Analysis

CLSM analysis was used to determine the effects of treatment on biofilm architecture. Specimens cultured with *E. faecalis* (1 × 10^6^ CFU/mL) in BHI broth with 1 μM of Alexa Fluor 647-labeled dextran conjugate (Molecular Probes, Eugene, OR, USA) for 7 days at 37 °C were divided into four groups (*n* = 6/group). Later, they were immersed individually into the experimental agents for 1 h each at 37 °C and 100% humidity. After treatments, the specimens were rinsed in DW and stained with 2.5 μM SYTO 9 (Molecular Probes, Eugene, OR, USA) at room temperature for 30 min. The images of the biofilms were obtained using an LSM 510 META CLSM microscope (Carl Zeiss, Jena, Germany). The thickness of the bacteria and EPS were quantified using COMSTAT (www.comstat.dk; Technical University of Denmark, Kongens Lyngby, Denmark) from ten image stacks (512 × 512 pixels) per experiment.

### 2.9. Field Emission Scanning Electron Microscope (FE-SEM) Observation

Morphological changes in biofilms following treatment were observed by FE-SEM. After treatment, the specimens were fixed in 2.5% glutaraldehyde (Sigma-Aldrich, St. Louis, MO, USA) and dehydrated using an ascending series of ethanol (25–100%). Then, the samples were sputtered with gold-palladium. Images of at least four randomly selected areas from each specimen were taken with the SU-70 FE-SEM (Hitachi, Tokyo, Japan).

### 2.10. NaOCl Treatment and CFU Counting

Thirty-six infected specimens were divided into two groups (control and experimental) (*n* = 18/group) after 7-days culture. Further, each group was divided into three (0%, 2.5%, and 5%) subgroups (*n* = 6/group). The specimens in the control group were pretreated with DW for 1 h, followed by treatment with varying concentrations of NaOCl (0%, 2.5% and 5%) for 1 min. The specimens in the 0% subgroup were treated with DW for 1 min. The specimens in the experimental group were pretreated with DNase (1 μg/mL) and melittin (6 μg/mL) for 1 h each, followed by treatment with varying concentrations of NaOCl (0%, 2.5%, and 5%) for 1 min. After treatment, the specimens were rinsed with 5% sodium thiosulfate to neutralize the NaOCl activity. Then, the remaining biofilms were harvested, serially diluted, and quantified as described earlier.

### 2.11. Statistical Analysis

The sample size was determined using G-Power 3.1 software (University of Düsseldorf, Düsseldorf, Germany). A power analysis with the F test (analysis of variance [ANOVA]) was applied, resulting in a required minimum sample sizes. To determine the normal distribution, the data were analyzed with the Kolmogorov–Smirnov test. Then, the statistical analysis was performed by using ANOVA, followed by Tukey’s test. These analyses were performed with the SPSS 12.0 software (SPSS Inc., Chicago, IL, USA). A *p*-value of <0.05 was considered significant.

## 3. Results

### 3.1. Antibiofilm Effect of Melittin and DNase on E. faecalis Biofilm

**Figure 1:**
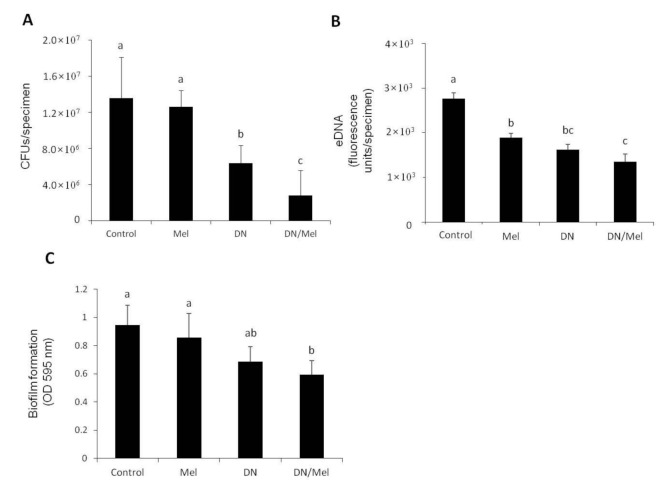
Evaluation of the antibacterial activity of melittin against *E. faecalis* biofilms. (**A**) Colony forming unit (CFU) counting. (**B**) eDNA measurement of *E. faecalis* biofilm. (**C**) Biofilm formation estimated spectrophotometrically using crystal violet staining. Values followed by the same superscripts are not significantly different (*p* > 0.05). Mel: melittin, DN: DNase, DN/Mel: DNase+melittin.

The MIC of melittin was determined as 6 μg/mL spectrophotometrically. Melittin reduced the CFU, but there was no significant difference with the control (*p* > 0.05). However, DNase-treated specimens exhibited 52% reduction in CFU. On the contrary, *E. faecalis* on specimens pretreated with DNase and then treated with melittin for 1 h each (DNase+melittin) showed a reduction of 79% in CFUs compared to control group (*p* < 0.05) (Figure 1A). A significant reduction in eDNA was noted in specimens treated with melittin, DNase, and DNase+melittin (*p* < 0.05), compared to the control. Notably, specimens treated with DNase/melittin showed the lowest eDNA levels (*p* < 0.05) (Figure 1B). Spectrophotometric analysis using crystal violet also showed the highest inhibitory effect in biofilm formation on specimens treated with DNase+melittin than those treated with either alone (*p* < 0.05) (Figure 1C).

**Figure 2:**
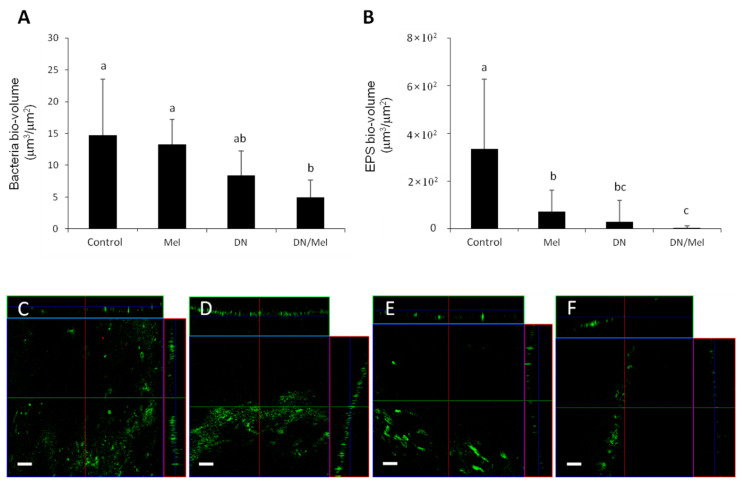
Determination of bio-volume of bacterial cells and extracellular polymeric substances (EPS) through confocal laser scanning microscopy (CLSM) analysis. (**A**) Bacteria bio-volume, (**B**) EPS bio-volume, and (**C**–**F**) representative CLSM images of *E. faecalis* biofilms grown on specimens. (**C**) Control (**D**) melittin, (**E**) DNase, and (**F**) DNase/melittin. Values followed by the same superscripts are not significantly different (*p* > 0.05). Mel: melittin, DN: DNase, DN/Mel: DNase+melittin. Scale bar = 100 µm.

**Figure 3:**
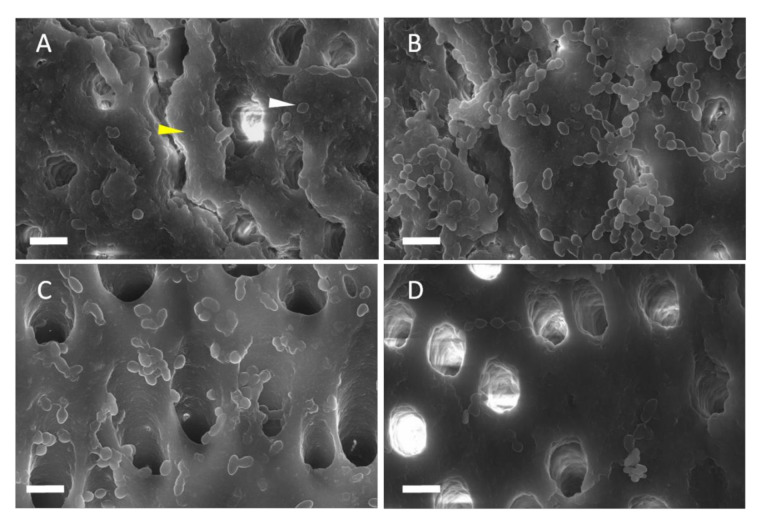
Representative field emission scanning electron microscope (FE-SEM) images of *E. faecalis* biofilms on specimens. *E. faecalis* was cultured in brain–heart infusion (BHI) for 7 days; (**A**) control, (**B**) melittin, (**C**) DNase, and (**D**) DNase/melittin. The yellow and white triangles indicate extracellular polymeric substance and bacteria, respectively. The magnification of the images was ×5000. Scale bar = 2 µm.

CLSM analysis showed that the combination of DNase+melittin significantly reduced the biomass compared to control (Figure 2A). Despite when there was no significant difference in CFUs, melittin reduced the EPS bio-volume significantly, implying that it weakens the structural stability of the biofilm matrix and thereby not by killing the bacteria (Figure 2B). SEM observations showed that DNase and DNase+melittin-treated specimens formed more disintegrated biofilms than the control and melittin. However, the bacterial cells in the DNase+melittin-treated samples were more distinguishable than the cells in the DNase treated group. A greater cell-free region exposing more dentinal tubules is observed in samples treated with DNase+melittin (Figure 3A–D). All these analyses confirm that specimens treated with DNase+melittin possessed a higher inhibitory effect on *E. faecalis* biofilms than melittin or DNase alone.

### 3.2. Susceptibility of E. faecalis Biofilm to NaOCl Following DNase+Melittin Treatment

**Figure 4:**
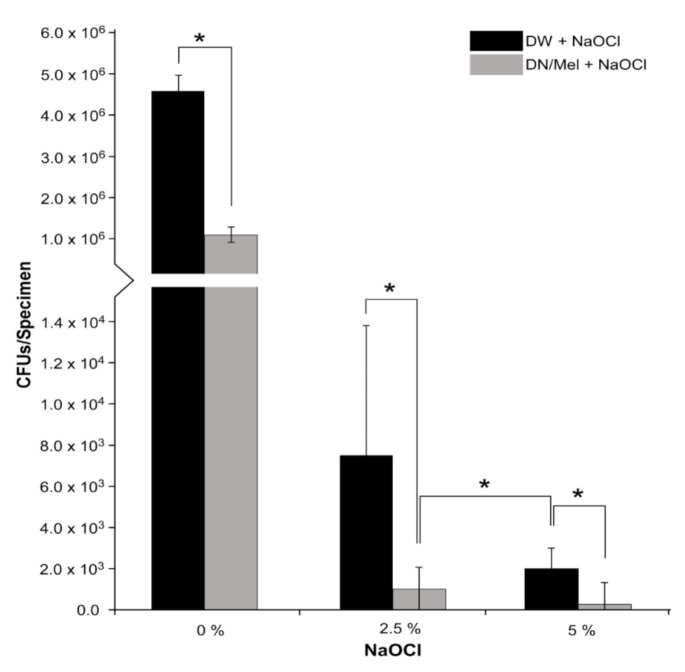
Synergistic effect of NaOCl with DNase and melittin (DN/Mel). Statistically significant *p* < 0.05 (*). DW: distilled water, DN: DNase, Mel: melittin, NaOCl: sodium hypochlorite.

The CFU assay indicated that the specimens pretreated with DNase (1 μg/mL) followed by melittin (6 μg/mL) for 1 h each showed higher susceptibility to 2.5% and 5% NaOCl with 98% and 99% reduction, wherein the specimens treated only with the same concentrations of NaOCl showed 86% and 95% reduction, respectively (Figure 4). Especially, 2.5% NaOCl on specimens pretreated with DNase+melittin showed lesser CFUs (98%) than specimens treated only with 5% NaOCl (95%) in 1 min (*p* < 0.05).

## 4. Discussion

Melittin, a potent amphiphilic cationic peptide, possesses antimicrobial, anti-tumor, and anti-inflammatory properties [22]. Numerous studies have highlighted the biomedical applications of melittin, but no studies have evaluated the antibiofilm effect against *E. faecalis* biofilms. To our knowledge, this present study evaluated the efficacy of melittin against *E. faecalis* biofilms for the first time.

According to this study, the MIC of melittin was 6 μg/mL. This suggests that melittin shows potent antibacterial activity against the planktonic form of *E. faecalis*. However, in CFU assay, when *E. faecalis* biofilm was treated with melittin, the number of bacteria was reduced but not significantly when compared to the control (Figure 1A). This might be due to the inability of melittin to penetrate through the biofilm matrix and act on the bacteria directly. Even when melittin was not able to reduce the CFUs, it significantly reduced the eDNA and EPS bio-volume (Figure 1B and Figure 2B). This could suggest that melittin exhibits its antibiofilm potential by reducing the eDNA and EPS bio-volume, which are responsible for biofilm stability and thereby weakening the biofilm matrix and not by killing the bacteria within the biofilm. A recent study also showed that the melittin down-regulated biofilm-associated protein (BAP) gene responsible for the EPS bio-volume in *Acinetobacter baumannii* [23]. This suggests that melittin could disrupt the biofilm matrix, making the bacterial cells more susceptible to NaOCl. Furthermore, numerous studies have established that the antibacterial activity of melittin was enhanced when combined with other antimicrobial agents [23,24,25].

To increase the antibacterial efficacy of melittin, combination with DNase was investigated to eradicate *E. faecalis* biofilms. DNase has been shown to sensitize *E. faecalis* biofilms to 2% chlorhexidine [7]. The eDNA formed as a by-product of cell lysis provides structural stability to the biofilms and protection against antimicrobials. Cleavage of eDNA by DNase could promote the penetration of antibiotics and decrease biofilm biomass and CFU levels [26,27]. Thus, in this study, pretreating *E. faecalis* colonized dentin specimens with DNase (1 μg/mL) reduced the eDNA level and thereby facilitated the entry of melittin causing a significant reduction of CFU (7.3 to 79%) and biomass (Figure 1A and Figure 2B). The FE-SEM images in the current study also revealed that specimens treated with DNase+melittin showed a higher degree of biofilm disruption, and more cell-free zones were observed compared to specimens treated with melittin or DNase (Figure 3A–D). In this respect, it is evident from the results that in the presence of DNase, melittin may be able to act more directly on *E. faecalis*, thereby enhancing its antibacterial and antibiofilm potential. A recent study showed a significant reduction in the CFU of planktonic *E. coli* and *S. aureus* following treatment with melittin in combination with graphene oxide [24].

The clinical application of melittin remains very challenging due to its cytotoxicity and high cost. Studies have been carried out on melittin modifications using nano-engineering to reduce cytotoxicity and cost [28]. For instance, the high manufacturing cost of melittin has been reduced by forming hybrid analogs with cecropin-A and melittin, which show higher antibacterial activity without inducing cytotoxicity [25]. In a similar attempt, graphene was conjugated with melittin to improve its antibacterial activity at low concentrations without inducing cytotoxicity [24]. It was shown in a previous study that melittin exhibited cytotoxicity at concentrations >10 µg/mL over 60 min exposure on human umbilical vein endothelial cells [29]. In this study, only 6 µg/mL melittin for 1 h was used in an attempt to reduce the cytotoxicity and cost. However, further studies should be carried out to evaluate the cytotoxicity of melittin to proceed onto clinical applications. Neither the DNase nor the decay products produced as a result of eDNA digestion possess toxicity, as the DNase does not penetrate cells and cleave only the eDNA [27]. Though DNase is a cost-intensive enzyme [30], the cost per usage when we use lower concentration is affordable in clinical practice. For instance, in this study, we showed that 1 µg/mL could efficiently act on *E. faecalis* biofilm. Thus, the proposed DNase+melittin complex could be a favorable approach to enhance the antibiofilm effect of melittin against *E. faecalis* biofilm at a low peptide concentration, which is a crucial step in decreasing the cytotoxicity and cost of natural peptide and helps in their future applications. According to the manufacturer’s instructions, both melittin and DNase should be stored at −20 °C for a longer shelf life of 2–3 years and should be relatively stable for 24 h at room temperature.

NaOCl at 5.25% concentration is caustic and reduces the elastic modulus and flexural strength of dentin [31]. Therefore, there have been attempts to develop less toxic alternatives [32]. This study showed that 2.5% NaOCl exhibited significant bacterial reduction when used with DNase+melittin complex than 5% NaOCl alone in 1 min (Figure 4). This might be due to the dissolution of the biofilm matrix by the DNase+melittin pretreatment, allowing better penetration and antimicrobial action of NaOCl. In this study, NaOCl was used after DNase+melittin treatment with intermediate water rinse to prevent any effects of NaOCl on the amino groups in the peptide [33]. It has been shown that 6% NaOCl killed 70% of the bacteria in the biofilms when treated with EDTA and peptide DJK-5 in 6 min [34]. By contrast, this study revealed that DNase+melittin, followed by 2.5% NaOCl for 1 min, reduced 98% of the bacteria in the biofilm. Within the limitations of this study, only monospecies biofilm was involved. Considering that endodontic infections can be polymicrobial, further studies should evaluate multispecies biofilms. Furthermore, general oral environment can be altered after prosthodontic [35], orthodontic [36], or implantologic [37] treatments. In these cases, the *E. faecalis* biofilm could be altered or modified. Therefore, further research is needed about the topic.

We used bovine teeth in this study, because they have several advantages compared with human teeth. First, a sufficient number of intact incisors can be obtained. Second, ethical issues regarding human-derived objects can be avoided. Furthermore, bovine root canals have wide surfaces; therefore, a standardized sample can be easily obtained. Therefore, bovine teeth have been widely used as specimens for endodontic microbiological studies [19,21,38,39,40].

## 5. Conclusions

Pretreating the biofilms with DNase improved the antibiofilm efficacy of melittin, and the combination of DNase+melittin demonstrated significant activity against *E. faecalis* biofilms. This could serve as an adjunct in potentiating the antimicrobial action of NaOCl significantly while reducing the concentration as well as the time of contact. This could help us to decrease the deleterious effect of NaOCl on dentin and periradicular tissues. Therefore, the null hypothesis was rejected.
